# Psychometric properties of the Persian version of delivery fear scale (DFS) in Iran

**DOI:** 10.1186/s12884-021-03634-7

**Published:** 2021-02-18

**Authors:** Aazam Shakarami, Mina Iravani, Mojgan Mirghafourvand, Mohammad Asghari Jafarabadi

**Affiliations:** 1 MSc of Midwifery, Midwifery Department, Reproductive Health Promotion Research Center, Ahvaz Jundishapur University of Medical Sciences, Ahvaz, Iran; 2 Midwifery Department, Reproductive Health Promotion Research Center, Ahvaz Jundishapur University of Medical Sciences, Ahvaz, Iran; 3 Midwifery Department, Social Determinants of Health Research Center, Tabriz University of Medical Sciences, Tabriz, Islamic Republic of Iran; 4 Road Traffic Injury Research Center, Faculty of Health, Professor of Biostatistics, Tabriz University of Medical Sciences, Tabriz, Islamic Republic of Iran

**Keywords:** Delivery, Delivery fear Scale, Fear, Iran, Psychometric

## Abstract

**Background:**

The aim of the present study was to evaluate the psychometric properties of the Persian version of delivery fear scale (DFS) among Iranian women population.

**Methods:**

This is a methodological study that was conducted to evaluate the psychometric properties of DFS. Convenience sampling was used to select 200 pregnant women from the maternity ward of Razi Hospital in Ahvaz, Iran. In the first step, the scale was translated into Persian using backward-forward translation method. Afterwards, the following types of validity were examined: face validity based on impact score, construct validity based on confirmatory factor analysis (CFA), and concurrent validity. The Pearson correlation test was used to determine the correlation of DFS with pregnancy-related anxiety questionnaire (PRAQ), Childbirth Attitude Questionnaire (CAQ), Spielberger’s state-trait anxiety inventory (STAI), and the short form of Lowe’s childbirth self-efficacy inventory. Reliability of DFS was assessed by determining internal consistency (Cronbach’s alpha) and split-half method.

**Results:**

CFA had satisfactory validity considering x^2^⁄df < 5 and the RMSEA < 0.08. /the obtained Cronbach’s alpha coefficient was 0.77. The split-half coefficient of the questionnaire was 0.83, indicating an acceptable reliability for the questionnaire. The results showed that DFS had a direct significant correlation with the CAQ (*r* = 0.72), PRAQ (*r* = 0.74), STAI-Y1 (r = 0.71) and STAI-Y1 (*r* = 0.63) and a reverse significant correlation with subscales of the short form of Lowe’s childbirth self-efficacy inventory including outcome expectancy (*r*= -0.75) and self-efficacy expectancy (*r*= -0.76).

**Conclusions:**

The findings of the present study confirm the validity and reliability of the Persian version of DFS as an instrument for measuring fear of childbirth (FOC) in Iranian women population.

## Background

Pregnancy and childbirth are one of the most important and wonderful experiences in most women’s lives. However, these experiences may be accompanied by severe stress and fear of childbirth for some women [1], which can lead to problems that can be solved by relying on the knowledge and skills of a variety of medical sources [2].

Fear of childbirth is referred to as tocophobia [3] which can present before pregnancy, after a traumatic or difficult delivery, or as a symptom of postpartum depression [4]. Fear of childbirth ranges from severe fear to rational fear of childbirth, with the latter being controlled during pregnancy [5]. However, the former is manifested in form of daily anxieties, nightmares, and physical symptoms, often leading to elective caesarean delivery upon maternal request [6].

Fear of childbirth affects about 6 to 10 % of women worldwide [7]. The prevalence of FOC was also 7–22 % in C-section deliveries in Finland, Sweden, and the United Kingdom [8]). Similarly, 93 % of the Iranian parturient women suffer from FOC [9]. FOC is a multi-factorial phenomenon that can be related to multiple psychological and social factors such as maternal beliefs, personality factors [10, 11], interaction between mothers and the medical team [12], fear of emergency interventions [13], labor injuries, inadequate support, loss of control, loneliness, and loss of a child [14].

Fear of childbirth can impose a negative effect on the experience of childbirth [15] and cause a more severe fear at subsequent deliveries [16]. It can also lead to prolonged and instrumental delivery [17]. This type of fear triggers postpartum depression, impaired mother-infant relationship, and emotional imbalance [18]. High FOC levels increase the risk of elective and emergency C-sections, and bring about abnormal patterns in fetal heart rate, low Apgar score, stillbirths [19], and low birth weight [20]. In addition, severe labor pain and fear in the active phase of labor can increase serum catecholamines and cortisol levels, which in turn leads to reduced uterine contractile force and uncoordinated contractions, eventually resulting in prolonged labor [21]. Furthermore, the stimulation of the sympathetic system also leads to increased cardiac output, hypertension, and elevated oxygen consumption, which in turn leads to increased risk of difficult childbirth and instrumental delivery [22], irritability, and fetal restlessness [23].

One of the negative consequences of FOC is the growing demand for caesarean section (C-section) [24]. Obviously, C-section is used as a last resort strategy to protect the life of the mother and baby in case of difficult deliveries which account for 10–15 % of the demand for C-section. Approximately, 85–90 % of deliveries can usually be performed normally without the need for any medical intervention [25]. The rate of C-section varies widely among different countries. That is, between 1980 and 2010, the rates were 20 % in Sweden, 20–30 % in the UK, and more than 30 % in the United States, while the rate was 46.2 % in China in 2008 [26, 27]. This growing trend has also been observed in Iran, with some studies reporting this rate to be around 50–60 %, which is 3–4 times more than the rate recommended by world health organization (WHO) [13, 28]. Iran was also the second leading country in terms of demand for C-section delivery in 2010 (42 %) [29]. By contrast, C-sections accounted for only 5 % of deliveries in the US in 2011 [8].

The results of a study conducted in Iran in 2010 revealed that the rate of C-section upon maternal request increased by 50 % due FOC, which was two times more than that in developed countries [1, 30]. However, researchers have shown a growing interest in addressing FOC and reducing its prevalence in the last decade. Since FOC leads pregnant mothers to opt for C-section delivery as mentioned above, it is essential to identify and measure FOC in pregnant women based on a valid scale. There are currently few instruments for measuring FOC in different communities. A case in point is Wijma Delivery Expectancy/Experience Questionnaire (WDEQ) designed by Wijma in two versions (A, B) in the late 80s. This instrument is used to measure FOC before and after delivery [31]. Childbirth attitude questionnaire (CAQ) is another instrument to measure FOC [32].

To assess fear during delivery, the Delivery Fear Scale (DFS) was developed by Wijma, Alehagen and Wijma (2002). The DFS is a practical scale that can be implemented in a very short time, and it has been shown to have a good psychometric quality in different cultures [25, 33]. However, the psychometric properties of the DFS have not been yet assessed in Iran. Therefore, the need for a culturally sensitive, reliable and valid instrument to be used to assess Iranian women’s fears during delivery is indispensable. Thus, given the importance of FOC and its impact on labor as well as maternal and neonatal outcomes, on the one hand, and the need for a standard adaptation of an existing scale for FOC measurement among Iranian pregnant women, on the other, the present study aimed to investigate the psychometric properties of the Persian version of DFS.

## Methods

### Study design

This is a methodological study that was conducted to evaluate the psychometric properties of DFS.

### Participants

The participants of this study were 200 pregnant women from the maternity ward of Razi Hospital in Ahvaz, Iran.

### Eligibility criteria

The study included pregnant women planning to have vaginal delivery, with gestational age equal to or more than 37 weeks, not having pregnancy-related risk factors (multiple pregnancy, preeclampsia, infertility treatments, etc.), expecting a healthy and live birth, willing to participate in the study, undergoing no general anesthesia, and not having any mental disability.

### Sample size

Selection of 10 samples per item for factor analysis is suggested by Nunnally and Bernstein [34]. Since there are 10 items in DFS, 100 samples are needed. In order for the tool to be applicable for both primiparous and multiparous women, 100 nulliparous women and 100 multiparous women (200 women in total) were selected.

### Sampling

Sampling was performed in the maternity ward of Razi Hospital in Ahvaz. A total of 200 pregnant women (100 primiparous and 100 multiparous pregnant women) were selected using convenience sampling.

### Recruitment

The researcher referred to the hospital, attended the maternity ward and examined the women admitted to the labor room for eligibility criteria. If they met the inclusion criteria, the objectives and method of the study were explained to them, and a written informed consent was obtained from them if they were willing to participate in the study. Data were then collected using questionnaires including demographic-obstetrics information, DFS, Pregnancy-Related Anxiety Questionnaire (PRAQ), Spielberger’s State-Trait Anxiety Inventory (STAI), Childbirth Self-efficacy Inventory (CBSEI), and Hartman’s CAQ through interviews.

### Instruments

#### **1. Questionnaire for demographic and obstetrics data collection**

This questionnaire included questions on the participants’ education level, occupation, income status, wanted or unwanted pregnancy, gravidity, and obstetrics history. The validity of this questionnaire was confirmed using qualitative content validity.

#### 2. Wijma’s delivery fear scale (DFS)

This questionnaire was designed by Wijma in 2002 to measure FOC [31]. DFS is a self-assessed questionnaire consisting of 10 questions with five items having positive meaning [1, 3, 5, 7, 10] and the remaining five items having negative meaning [2, 4, 6, 8, 9]. The questions are scored based on a 10-point Likert scale ranging from (Strongly disagree = 1 to strongly agree = 10). The positive items are scored in reverse. The possible score range is from 10 to 100 that is calculated by summing up the total scores of the questionnaire. Higher scores indicate greater FOC. This scale has an acceptable reliability (α = 88 %) [25].

#### 3. Spielberger’s State-Trait anxiety inventory (STAI)

This questionnaire was designed in 1970 to measure state and trait anxiety [35]. This inventory consists of 40 questions, 20 of which are related to state anxiety (expressing personal feelings) and the remaining 20 questions measure the trait anxiety (general and common emotions). This scale is scored based on a 4-point Likert scale, ranging from 1 (lowest level of anxiety) to 4 (highest level of anxiety). Items 1, 2, 5, 8, 10, 11, 15, 16, 19, 20 (state anxiety scale) and items 21, 23, 26, 27, 30, 33, 34, 36, 39 (trait anxiety scale) are scored in reverse. The possible score range is from 20 to 80 for each scale, with scores 20–31,32–42, 43–53,54–64, 65–75, and 76 and above indicating mild, moderate to low, and moderate to high, and relatively severe, severe, and very severe anxiety, respectively. The reliability of this questionnaire was confirmed based on previous studies in Iran with Cronbach’s alpha coefficient of 0.84 and content validity [9].

#### 4. Short form of Lowe’s childbirth self‐efficacy inventory

This inventory was designed in 1993 by Lowe [36]. It consists of 34 questions in two sections. The first section contains 17 questions that measure outcome expectancies. The second section also includes 17 questions that measure self-efficacy expectancies. Questions are scored based on a 10-point Likert scale and the possible score range is from 17 to 170, with higher scores indicating higher outcome expectancy and more self-efficacy expectancy. This inventory enjoys a high internal consistency (0.84–0.91) [37].

#### 5. Short form of Van den Berg’s pregnancy-related anxiety questionnaire (PRAQ)

This questionnaire was designed by Van den Berg in 1989 to measure pregnancy-related fears and concerns. The short form of this questionnaire consists of 17 items. Exploratory factor analysis of the data of this questionnaire revealed five factors: Factor 1: Fear of Childbirth (3 questions), Factor 2: Fear of giving birth to a physically or mentally handicapped child (4 questions), Factor 3: Fear of change in marital relationships (4 questions), Factor 4: fear of changes in mood and its effects on the child (3 questions), and Factor 5: self-centered fears or fear of changes in the mother’s personal life (3 questions). The final score is obtained by adding up the scores of each statement. Each score is rated based on a 7-point Likert scale, and the possible pregnancy-related anxiety scores range from 17 to 119 [38]. The psychometric properties of this questionnaire have been reviewed and approved by Karamoozian et al. (2016) in Iran. Its reliability was also confirmed (α = 0.78) and ranged from 0.69 to 0.76 for each of the five factors [39].

#### 6- Childbirth Attitude Questionnaire (CAQ)

Childbirth Attitude Questionnaire (CAQ), developed by Hartmann (1988) [40], consisted of 16 questions, and was revised by Louis. It is used to measure fear of childbirth. Questions are answered based on a 4-point Likert scale (Not at all = 1, Very low = 2, Moderate = 3, High = 4). The possible score range is from 14 to 56, with higher scores indicating higher FOC. Given that there was no cut-off point for FOC, similar international studies were considered to determine the cut-off point, and the median score (i.e. 28 or more) was considered as FOC. Cronbach’s alpha coefficient was used to determine the internal consistency (α = 0.83) [32].

### Translation process

At first, the researchers obtained official permission from the main designer of the questionnaire (Wijma) for evaluating the psychometric properties of the questionnaire in Iran. The original version of the questionnaire was translated from English into Farsi by two experts in English and reproductive health. The Farsi version was then translated back into English by two translators fluent in both languages who had not read the original version. The two translations were then compared, the questions were matched in terms of meaning and concept, and finally an approved version was prepared. Attempts were made to ensure the accuracy of translation and comprehensibility of questions.

### Face and Content Validity

To determine face validity, a qualitative method was used. To this aim, 30 randomly selected pregnant women were asked to rate all questions in terms of difficulty, relevance, and ambiguity. Then based on the comments provided, the questionnaire was modified, if necessary.

Content validity was also assessed by a qualitative method. For this purpose, 10 midwifery and reproductive health specialists were asked to examine the translation of each question in terms of grammar, vocabulary use, and correct placement of statements and to provide their corrective comments [41].

### Construct validity

Construct validity refers to the ability of the instrument to measure the target concept or conceptual structure. Factor analysis is a common method for evaluating construct validity [42]. In this study confirmatory factor analysis (CFA) was performed to evaluate the construct validity. Fit indices were used to evaluate the suitability of the EFA model. To confirm the model, the following indices were used: root mean square approximation (RMSEA) less than 0.08, standardized root mean square error of approximation (SRMSEA) < 0.08, comparative fit index (CFI) ≥ 0.90, tucker-lewis index (TLI) ≥ 0.95, and normed crystal (x^2^ / df) < 5.0.

### Concurrent validity

In order to evaluate the concurrent validity of DFS in the present study, the correlation of the tool was assessed with other valid tools using convergent and divergent validity. Convergent validity means the degree of correlation between scores from one instrument and those from another. The higher the correlation, the greater the convergent validity of that instrument. Convergent validity was also assessed using PRAQ, STAI and Hartmann’s fear of childbirth scale. The researcher then calculated correlation between the scores of these questionnaires with DFS. Divergent validity means the degree of distinction between scores from one instrument and those from another. Again, the higher the correlation, the greater the divergent validity of that instrument. Divergent validity was also assessed using the short form of Lowe’s childbirth self-efficacy inventory. The researcher then calculated the correlation between the scores of this questionnaire and those of DFS. Values ​​of 0.8-1, 0.5–0.8, 0.3–0.5, 0.1–0.3, and 0-0.1 represent very strong, strong, moderate, poor, and low correlation, respectively.

### Reliability

Internal consistency and split-half methods were used to determine the reliability of the questionnaire. Cronbach’s alpha coefficient was calculated to determine internal consistency which examines the degree of correlation between the variables that make up the scale in question. In the present study, the Cronbach’s alpha value should be greater than 0.7 to obtain an acceptable reliability [43].

In the split-half method, the best way was to include all odd questions in one test and all even questions in another test. Accordingly, 30 women admitted to the maternity ward of Razi Hospital of Ahvaz who were in active phase of labor and met the inclusion criteria were selected by simple random sampling and completed the questionnaire. The questionnaire was then divided into two equal halves and each half was scored separately. The correlation coefficient between scores of each split-half-test was calculated using Spearman-Brown formula. An index higher than 0.7 indicated optimal stability.

### Statistical analysis

Statistical analysis was performed in SPSS25 using a Windows device (IBM Inc., Armonk, NY, USA) and Stata15 (StataCorp, College Station, USA). To describe the participants’ characteristics, the frequency was used for binary variables, and mean (standard deviation).

was used for continuous variables.

### Ethical consideration

The study was approved by the Ethics Committee of Ahvaz University of Medical Sciences (Ref. No.: IR.AJUMS.REC.1397.720). Before using the FDS, the required permission was obtained from Prof. Klaas Wijma via email. Participants were informed of the objectives of the study and signed a written informed consent form. Also, they were free to withdraw from the research at any time.

## Results

A total of 200 pregnant women were enrolled in the study between May and July 2019. The mean (SD) of participants’ age was 27.2 (6.2) years. Most of them (92 %) were housewives and had high school education (71 %) and middle income (83 %) (Table 1).

**Table 1:** Characteristics of the study participants (*N* = 200)

Characteristics	Mean (SD^a^)
Age (Years)	27.2 (6.2)
	**Number (Percent)**
Education	
Intermediate or below	32 (16)
Diploma and high Scholl	141 (71)
College	27 (13)
Job	
Housewife	184 (92)
Employee	26 (8 )
Income	
Not at all sufficient	40 (21)
Relatively sufficient	83 (41)
Completely sufficient	77 (38)

^a^Standard Deviation

### Construct Validity

**Fig. 1:**
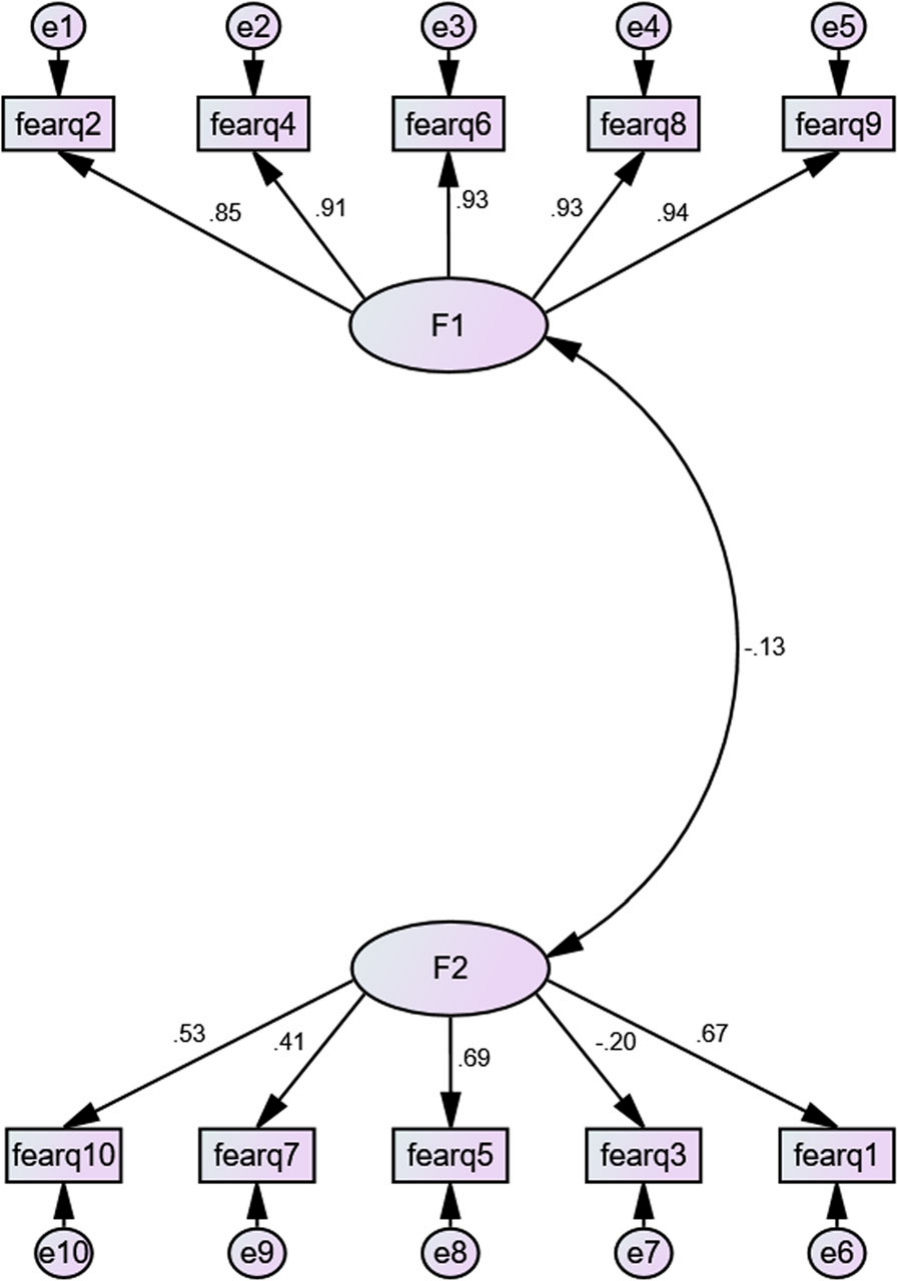
Factor structure model of the FDS

According to the values ​​of the indices presented in Table 2 (CFA), chi-square/df ratio < 5 and the RMSEA index value < 0.08, RMR index ​​< 0.1 were obtained, which confirmed the reliability of this model. The fit of GFI, AGFI, NFI, TLI, RFI, IFI and CFI was greater than 0.9. Therefore, this model obtained an optimal level of fit, and the factor structure can be confirmed, accordingly. As a result, the Persian version confirmed the original version of questionnaire as a two-factor structure. Figure 1 shows the standard coefficients of CFA for this scale.

**Table 2:** Confirmatory factor analyses fit Index of the Delivery Fear Scale (DFS) (n = 200)

Fit Indices	Fit
χ2	41.888
df	34
P	0.166
$$\raisebox{1ex}{${x}^{2}$}\!\left/ \!\raisebox{-1ex}{$df$}\right.$$	1.232
GFI	0.959
AGFI	0.934
NF1	0.968
RF1	0.958
IF1	0.994
TLI	0.992
CF1	0.994
RMSEA (90 % CI)	0.034 (< 0.001, 0.065)

χ2: chi-square; df: degrees of freedom; *χ2/df* normed chi-square; *RMR* Root Mean R; *GFI* Goodness of Fit Index; *AGFI *Adjusted Goodness of Fit Index; *RMSEA* root mean square error of approximation; *NFI* Normed Fit Index; *RFI* Relative Fit Index; *IFI* Incremental Fit Index; *TLI* Tucker-Lewis Index; *CFI* Comparative Fit Index

### Concurrent validity

The results showed that DFS had a direct significant correlation with the CAQ (*r* = 0.72), PRAQ (*r* = 0.74), STAI-Y1 (*r* = 0.71) and STAI-Y1 (*r* = 0.63). Also, DFS had a reverse significant correlation with subscales of short form of Lowe’s childbirth self-efficacy inventory including outcome expectancy (*r*= -0.75) and self-efficacy expectancy (*r*= -0.76) (Table 3).

**Table 3:** The results of the concurrent validity of the questionnaires (*n* = 200)

Scale	Significance level	Correlation coefficient
State-Trait Anxiety Inventory, Form-Y1	< 0.001	0.71
State-Trait Anxiety Inventory, Form-Y2	< 0.001	0.63
Pregnancy-Related Anxiety Questionnaire	< 0.001	0.74
Childbirth Attitude Questionnaire	< 0.001	0.72
Childbirth Self-Efficacy Inventory-Outcome expectancy	< 0.001	-0.75
Childbirth Self-Efficacy Inventory-Self-efficacy expectancy	< 0.001	-0.76

### Reliability

The questionnaire had optimal Cronbach’s alpha coefficient (α = 0.77). Item three had a Cronbach’s alpha value lower than 0.3. Also, the split-half reliability coefficient was calculated to be 0.83 (Table 4).

**Table 4:** Reliability Statistics of the Delivery Fear Scale (DFS) (*n* = 200)

Number of Items	Cronbach’s Alpha	Split-half coefficient
10	0.770	0.83

## Discussion

The aim of the present study was to evaluate the psychometric properties of DFS in Iranian pregnant women. The results showed that the Persian version of DFS is a reliable and valid instrument for assessing FOC among this particular population.

All items of DFS had an impact score higher than 3. The results showed the clarity, simplicity, and relevance of the items.

Confirmatory factor analysis was performed to determine the construct validity. The results of the chi-square test showed a significant difference among the covariance matrices examined in our study (*P* > 0.05). This indicates that the observed matrix is ​​consistent [44].

The results of chi-square/df ratio were also evaluated. A chi-square/df ratio below 3 indicates good fit and a chi-square/df ratio < 5 indicates moderate fit. In this study, X^2^ (SD = 28/835) indicated suitable fit. In the study of Serçekuş et al., X^2^ (SD = 31/6335) was reported to indicate good fit [25]. In the present study, the RMSEA was < 0.08, indicating good fit. Consistent with the present study, Serçekuş et al. reported RMSEA = 0.00. Also, GFI, AGFI, NNFI, and CFI indices show good fit when their value is more than 0.9 and 0.94 [45]. In the present study, GFI, AGFI, NNFI, CFI values were > 0.9, indicating good fit for the scale. In Serçekuş et al.’s study, GFI, AGFI, NNFI, CFI were equal to 0.94, indicating good fit.

The present study demonstrated a significant positive correlation of STAI, PRAQ and Hartman’s fear of childbirth with DFS, and a significant inverse correlation between DFS and short form of Lowe’s childbirth self-efficacy inventory, which supports its concurrent validity. Concurrent validity refers to the extent to which the findings of a measurement, or particular test, correspond to those of a previously approved measurement for the same construct [46]. According to the correlation obtained in the present study, DFS has good concurrent validity. Similar to our study, Serçekuş et al. found a significant, positive correlation between the state anxiety inventory and DFS (r = 0.80) [25].

Cronbach’s alpha coefficient was calculated for constructs of the DFS in Iran (0.77), which indicates an optimal internal consistency. The results are consistent with the original version (0.88) [31]. In other studies, this scale had a Cronbach’s alpha coefficient of 0.91, which is similar to the results of Wijma et al. [25, 47] but different from that of Kim et al.’s study, (α = 0.72). This difference can be due to inter-cultural differences [33]. The split-half coefficient was 0.83 for the scale, which confirmed the reliability of the present study. Serçekuş et al. also obtained a split-half coefficient of 0.81 [25].

### Implications for practice

The consequences of the fear of childbirth are a widespread problem affecting childbirth and the postpartum period. Therefore, it is important to take appropriate interventions for women with FOC. Results of the present study revealed that DFS is a valid instrument that can be used for Iranian women. DFS is a practical and quick instrument for determining the FOC level. Therefore, it can be implemented in a very short time. It is recommended that DFS be used to determine the FOC level in women during childbirth. Diagnosing FOC may help the pregnant woman have a better delivery experience through ongoing supportive care (emotional support, information, relaxation, etc.).

It is hoped that development of a psychometrically valid and reliable questionnaire will lead to identification of Iranian pregnant women with real pathological fear and then to elevate their awareness and self-confidence to contribute to the process of normal childbirth, reduce cesarean rates, and improve maternal and neonatal outcomes with effective educational and counseling interventions during pregnancy and labor. It should be noted that the translation and adaptation of a scale in different languages ​​makes it possible to use that scale in multicenter and international studies.

### Strengths and limitations

One of the strengths of the present study was the determination of divergent validity of DFS questionnaire using the short form of Lowe’s childbirth outcome expectancy self-efficacy inventory and its convergent validity using Van den Berg’s pregnancy-related anxiety questionnaire (PRAQ), Hartman’s fear of childbirth, and Spielberger’s state-trait anxiety inventory (STAI). However, our study had its own limitations, too. One limitation of the present study could be attributed to the concurrent investigation of nulliparous and multiparous women. Also, the fact that psychometric evaluation of the questionnaire was assessed only in Ahvaz with its own unique culture may limit the generalizability of the results. Therefore, it is recommended to repeat the psychometric evaluation of the same questionnaire in other parts of Iran with different cultures and in rural areas.

## Conclusions

The findings of the present study showed that the Persian version of DFS is a valid and reliable instrument for measuring FOC. This instrument will help specialists and medical staff to measure FOC in pregnant women in the delivery room and provide supportive interventions.

## Data Availability

The datasets used and/or analyzed during the current study are available from the corresponding author upon reasonable request.

## Abbreviations

DFS: Delivery Fear Scale
EFA: Exploratory factor analysis
CFA: Confirmatory Factor Analysis
PRAQ: Pregnancy-Related Anxiety Questionnaire
STAI: Spielberger's State-Trait Anxiety Inventory
FOC: Fear of childbirth
W-DEQ: Wijma Delivery Expectancy/Experience Questionnaire
CAQ: Childbirth Attitude Questionnaire
RMSEA: Root Mean Square Approximation
SRMSEA: Standardized Root Mean Square Rrror of Approximation
CFI: Comparative Fit Index
TLI: Tucker-Lewis Index
